# Childhood violence across distinct, overlapping, and concurrent contexts: polyvictimization, polyperpetration, and missed interventions points among child knife crime fatalities in England

**DOI:** 10.3389/fsoc.2026.1771547

**Published:** 2026-04-20

**Authors:** Jade Levell, Tom Roberts, Jo Staines, Vicky Sleap, Sylvia Stoianova, Edward Carlton, Karen Luyt

**Affiliations:** 1 School for Policy Studies, University of Bristol, Bristol, United Kingdom; 2 Department of Emergency Medicine, Bristol Medical School, University of Bristol, Bristol, United Kingdom; 3 National Child Mortality Database, Child Mortality Analysis Unit, Bristol Medical School, University of Bristol, Bristol, United Kingdom

**Keywords:** child death, child homicide, domestic violence and abuse, knife crime, polyperpetration, polyvictimization, school exclusion, serious youth violence

## Abstract

**Introduction:**

This study examines casefiles of children in England who were fatally stabbed between 2019 and 2024, focusing on their prior experiences of violence and adversity, aiming to identify missed opportunities for earlier intervention.

**Methods:**

We conducted a quantitative analysis using descriptive statistics and qualitative casefile analysis of 58 casefiles from the National Childhood Mortality Database, encompassing child fatalities by stabbing in England between 2019 and 2024, which had files available. Casefiles were examined for indicators of prior victimisation and perpetration. Patterns of polyvictimisation and polyperpetration were identified to understand concurrent and cumulative experiences of violence.

**Results:**

Casefile analysis revealed that, prior to this point, a significant number (58%) of children had experienced childhood domestic violence and abuse and were seldom supported by specialist services. 59% had experienced a victim/perpetrator overlap.

**Discussion:**

Casefile narratives indicated a range of barriers to effective support related to identification, assessment, and classification of early experiences of violence. Findings show that children are seldom treated as primary victims of childhood domestic violence and specialist support is often missed. Current intervention pathways fail to recognise the holistic, long-term, overlapping and cumulative experiences of violence in children’s lives.

## 1 Introduction

Drawing on retrospective case records held by the National Childhood Mortality Database (NCMD), this research explores the experiences of violence of 58 children who ultimately died from knife injuries. The aim of this study was to identify and examine the life trajectories of children prior to their deaths, investigating commonalities in experiences of violence and adversity, and the missed and offered intervention points for earlier support. To analyze the NCMD case records we utilized the dual lenses of polyvictimization (Turner et al., 2016) and polyperpetration (Schokkenbroek et al., 2024) to explore the distinct spatial domains (home, school, and community) that violence occurred in the children’s lives, both in prior experiences and the fatal injury. The analysis highlighted that many children experienced diverse types of violence in multiple domains prior to their early deaths, yet our current siloed support system often failed to appropriately recognize and support children at the point of disclosure, and when they did, they often failed to see the interconnected experiences across domains. Although it is not possible to conclusively say whether a specific intervention would have made a difference, it is plausible to believe that appropriate interventions that acknowledge the extent of violence cutting across different domains of home, school and community in some children’s lives could have made a positive difference.

In England, statutory death notifications are required to be submitted to one of the 58 regional Child Death Overview Panels, for any child who dies before their 18th birthday. The Children Act 2004 mandates that the Panels must carry out a review of the circumstances of death and the modifiable factors relevant to the death as part of the child death review (CDR) process. Since 2019, this data has been collated by the NCMD Programme, allowing detailed analysis of the circumstances of death at a local and national level. Identifying and understanding the factors that contributed to these deaths can help to ensure that appropriate preventive actions are established to reduce rates of child fatalities. The NCMD is the first national database of its kind to systematically collate information about the deaths of all children, from birth to 17 years old, and provides a unique insight into the lives of children who have died before reaching adulthood.

Using a sharp instrument is the most common method of homicide in the UK and has been since 1977 (Allen and Wong, 2025). The issue of knife crime in the UK has been gaining significant interest over the past decade due to an increase in knife related offences and deaths. Police-recorded offences involving a knife or sharp instrument between 2014 and 2024 have increased by 81%, with teenage knife related deaths increasing by 240% in the last decade (ONS 2025). Boys are most at risk of engaging in knife crime in the community, with girls more at risk of using knives at home (Browne et al., 2022). Knife crime is most likely to occur in and around schools in the UK (Bailey et al., 2020). The victim-perpetrator overlap is not uncommon. A 2025 survey of 11,000 young people in the UK found that in the past year, nearly one in five (18%) had been a victim of violence, one in eight (13%) had carried out violence themselves and half (50%) had witnessed violence being committed against someone else (Youth Endowment Fund, 2025). In this paper we have used the distinct lenses of polyvictimization and polyperpetration to make visible the ways in which, for many children, the fatal injury was not the first or only experience of violence. For instance, a child may be experiencing victimization at home, while simultaneously perpetrating violence at school. Expanding Turner et al.’ (2016) model of polyvictimization to also focus on polyperpetration enables a deeper understanding of the different types of violence children experience in different spaces (home, school, and community). This work builds on prior work examining the life paths of children who had experienced both domestic violence and gang violence in childhood (Levell, 2020). Using retrospective case file data about children who died from knife crime, this study aimed to explore the pre-injury factors present in children’s lives prior to their deaths and whether there were potentially missed opportunities to provide targeted support that may have changed the trajectories of their lives.

### 1.1 Adverse childhood experiences and the cumulative burden of childhood victimization

There has been attention to the short- and long-term impact of multiple forms of adversity in the lives of children for decades, popularized in the ‘adverse childhood experiences’ (ACEs) study (Lacey and Minnis, 2020). Felitti et al. (1998) conducted a large-scale survey in the USA which explored seven categories on a 10-point scale of ACE that they then associated with adult health risk behaviours and diseases. These include childhood experiences of violence, abuse, loss, and parental mental health issues, substance misuse, and imprisonment. It is commonly cited that being subject to four or more ACEs places individuals at greater risk of a range of negative health and wellbeing outcomes, such as violence, mental illness, and substance use, across the life course (Hughes, 2017). However, the use of cumulative ACE scores has been critiqued for being reductive and can obscure the different harms of distinct ACEs (Amos et al., 2023).

The experience of ACEs is correlated with both enhanced risks of later violence – both victimization and perpetration. For example, experiencing four or more ACEs is associated with a 15 times greater likelihood of having committed violence against another person in the past 12 months (Bellis et al., 2016). Fagan (2022, p 1722) found that ‘the number of ACEs experienced in childhood (ages 0-12) increased the likelihood of physical but not verbal violence (i.e., intimidation) or witnessing violence’. Research by Duke et al. (2010) into ACEs and adolescent violence perpetration indicated that ACEs substantially increased risks both of outward facing violence, such as intimate partner violence, bullying, fighting, weapon-carrying, as well as self-directed violence such as suicidality and self-harm. Unsurprisingly research has emphasized that ‘justice-involved children have childhoods characterized by disproportionate adverse childhood experiences’ (Gray et al., 2023, p. 4). These risks are often gendered, with boys particularly vulnerable to the negative effects of cumulative ACEs (Forster et al., 2020). The growing awareness of the adverse childhood experiences that are disproportionately faced by young boys who are ‘at risk’ of involvement in youth violence has been articulated by Baird (2024) as ‘chronic vulnerability’, which is intergenerationally transmitted and compounded by intergenerational poverty, trauma, and social exclusion. The higher levels of childhood adversity among adolescent boys who become involved in serious youth violence raises questions about enhancing prevention strategies.

## 2 Polyvictimization and polyperpetration

To examine this the theory of polyvictimization (being a victim of multiple forms of violence) and polyperpetration (being accused or convicted of multiple forms of harm against others) are helpful frameworks. The term ‘polyvictimization’ was first used by Finkelhor et al. (2007) who resisted a siloed approach to violence classification. It refers to an understanding of the complete profile of multiple victimization one may experience, as well as the cumulative burden of this (Hamby et al., 2018; Leffler and Spivak, 2018). A key issue for young people who experience polyvictimization of violence within different contexts is that they face systems which are overly siloed and fail to understand a holistic picture of violence in the child’s life across multiple domains (Turner et al., 2016; Hamby et al., 2018; Levell, 2020; Lee et al., 2023). It is distinct from ACEs discourse as it homes in on violence, rather than wider signs of adversity. Polyvictimization makes visible the experiences of diverse forms of violence, rather than focusing on multiple incidences of the same type of violence. This is complicated, however, by the fact that a single perpetrator may victimize a child in multiple ways, or there may be multiple perpetrators (Källström et al., 2020). Tura et al. also found that boys were more likely to experience poly-victimization than girls (Tura et al., 2023, 2026).

Research on polyperpetration has developed later largely focusing on violence and polyperpetration within contexts of intimate partner abuse (Jeensuk and Joonbeom, 2024; Schokkenbroek et al., 2024; Willie et al., 2017; Willie and Kershaw, 2018). Much of the research focuses on the prevalence of childhood experiences of adversity and trauma in the backgrounds of polyperpetrators, highlighting the intergenerational nature of violence involvement. Research suggests that experience of childhood maltreatment, including physical and sexual abuse and neglect, significantly increase the risks of children perpetrating violence themselves (Milaniak and Widom, 2015). Many children are entangled within a ‘web of violence’ (Hamby et al., 2018), often facing statutory systems that are overly siloed and which fail to understand the holistic picture of violence in the child’s life. Understanding children’s concurrent experiences of violence (as both victims and perpetrators) requires attention both to distinct typologies of violence experienced (for example, domestic violence or serious youth violence) as well as the distinct spatial domains in which they occur (home, school, community). Viewed through this lens can make visible a child who is a victim of violence at home but simultaneously perpetrating violence in the community, or vice versa thereby allowing more holistic interventions.

### 2.1 Gendered and intersectional aspects of the victim/perpetrator overlap

Polyvictimization and polyperpetration in childhood must be understood through an intersectional lens. Serious youth violence is highly gendered, with boys who face intersecting marginalization through race, class, and deprivation far more likely to be both victims and perpetrators (Williams et al., 2023). Recent research has emphasized the ways in which boys mediate their experiences of adversity and violence through the lens of masculinities in contexts of intergenerational vulnerability shaped by poverty, trauma, and exclusion (Baird, 2024; Gray et al., 2023; Levell, 2020; Maguire, 2021). Levell’s (2020) work on boys’ life trajectories shows that they often experience dual exposure to violence in private and public spaces yet are often invisible to domestic abuse services while being hyper-visible to youth justice systems. Living in a home where violence and abuse is present can push children and young people out of home/care and into environments that increase the risk of being targeted by exploitative peers or adults (Papalia et al., 2025). Children’s Commissioner (2019) found that children who were in gangs were 37% more likely to have experienced childhood domestic violence and abuse (DVA) than other children. Longitudinal evidence from the Millennium Cohort Study shows that children experiencing DVA in early childhood are more likely to carry a knife at age 17, increasing the risks of further violence to themselves and others (Villadsen and Fitzsimmons, 2021). Research into prevalence and predictors of adolescent polyvictimization in England and Wales found that committing a criminal offence was associated with a greater risk of becoming a polyvictim (Tura et al., 2023, p. 4702). Furthermore, estimates suggest that 50 to 80 per cent of young people who engage in child to parent abuse have prior experience of domestic abuse in the family home (Simmons et al., 2018). These findings underscore the need for services to recognize how early violence shapes later trajectories and intervene sooner.

## 3 Materials and methods

This study was based on a retrospective cohort review of data held by the National Child Mortality Database of 145 children who died through stab wounds, between April 2019 and March 2024. At the time of this analysis (November 2024), there were 58 cases where the final CDOP review was available, following the completion of court proceedings. The aim of the study was to explore the complexities of children’s concurrent experiences of diverse forms of violence, and the victim-perpetrator overlaps, by addressing the following questions:

- What types of violence (both victimization and perpetration) were recorded in the NCMD case records of children who died through stab wounds?
- What spatial domains of violence were recorded in the NCMD case records of children who died through stab wounds?
- What did the professional remarks in NCMD case files reveal about identification, referral, and support pathways for the children?

Each NCMD record consists of forms completed by professionals at the time of death, accompanied by any other official records kept on the child, which may include postmortem reports, Joint Agency Response meeting notes, relevant Police reports and/or Children’s Services records. This list is not exhaustive and varies case-by-case, which is a limitation of the data set (discussed further below).

The case files were analyzed using a mixed methods approach. The research team first used descriptive statistical analysis to help us identify broad patterns within the NCMD case files we examined. This approach enabled us to summarize key demographic features, risk factors and agency-involvement histories across cases, despite the variability in the level of detail found within case files. We were particularly mindful of the need for systematic coding, given evidence that publicly available CDR reports often contain inconsistent information and lack standardized reporting (Mantell et al., 2024). Our analysis was informed by national case review findings that have highlighted recurrent themes of childhood adversity and cumulative harm, and we saw similar patterns across the cases we reviewed (Garstang et al., 2021). Drawing on government-commissioned overviews of SCRs strengthened our understanding of broader trends in children’s vulnerabilities, the nature of the deaths and multi-agency contact prior to these incidents, reinforcing the value of descriptive statistics in identifying systemic issues and informing safeguarding improvements (Dickens et al., 2022).

To analyze the qualitative casefile material in depth, we undertook a structured, multi-layered coding process that combined deductive and inductive approaches. Pre-injury data were first manually coded using three distinct frameworks, each selected to illuminate different dimensions of children’s lived experiences. Our initial deductive stage involved coding each case for items within the Adverse Childhood Experiences (ACEs) framework (Felitti et al., 1998; Boullier and Blair, 2018). Because case records frequently referred to ‘neglect’ without specifying whether this was emotional or physical, we collapsed these into a single combined category to ensure consistency across files. Following this, we applied Turner et al.’s (2016) model of polyvictimization, which conceptualizes children’s experiences of harm across spatial domains (home, school, and community). This model enabled us to systematically identify the context in which violence or adversity had occurred, while also allowing us to extend the approach to examine polyperpetration, coding for the presence of perpetration across these same domains. This additional layer offered insight into the social ecology of harm and the complexity of children’s interactions with different actors in their environments.

Building on these deductive phases, we then undertook a narrative casefile analysis using inductive thematic coding (Labra et al., 2020). Through iterative reading and coding, we explored how children’s prior experiences of violence were documented, the extent to which concerns had been recognized by professionals, and whether referrals to services had been made. We also examined what kinds of interventions, if any, were subsequently offered. This inductive phase enabled us to capture patterns of service involvement, missed opportunities for intervention, and the broader structural or relational factors influencing children’s trajectories. Together, these three complementary coding frameworks provided a comprehensive and layered understanding of the children’s pre-injury experiences, the contexts of their victimization, and the systemic responses that shaped their care pathways. For consistency it was the same researchers who undertook the coding process. To ensure consistency and rigour, our research team worked collaboratively throughout the coding process, regularly sense-checking one another’s interpretations and cross-reviewing coded material to maintain reliability across researchers.

### 3.1 Ethical approval

The study was reviewed by the Chair of the Central Bristol NHS Research Ethics Committee, who confirmed that formal NHS ethical approval and individual consent were not required, as the work falls under Usual Practice in Public Health and Health Protection. However, it is essential to acknowledge that these casefiles represent the lives of children whose deaths caused profound grief and loss for their families, friends, and communities. We approach this work with deep respect for their memory and the hope that these findings contribute to preventing future tragedies.

### 3.2 Limitations

This research focused on a small sample of child deaths. The data held on each child within the NCMD is variable, being dependent on the child’s prior engagement with services and the data submitted to the NCMD. It is likely that incidents of DVA are under-represented within the casefiles, as the majority of DVA cases are not reported to the police or social services The child’s voice is largely absent from the records due to the nature of the reports and so there is an emphasis on professional narratives within the data. Nonetheless, the casefiles still shed light on the multiple overlapping experiences of violence and harm faced by these children before their deaths.

### 3.3 Details of the sample cohort

Table 1 provides demographic information for the full cohort of 145 children who died from knife wounds during the study period, and for the 58 children whose final CDOP review was available.

**Table 1:** Demographics of the full and detailed analysis cohorts.

Demographics	Number	Percentage	Number	Percentage
Age < 5 yrs.	9	6.2	5	8.6
5–10 yrs.	9	6.2	3	5.2
11–14 yrs.	12	8.3	6	10.3
15–17 yrs.	115	79.3	44	75.9
Missing	0	0	0	0
Sex				
Male	131	90.3	54	93.1
Female	13	9.0	4	6.9
Missing	1	0.7	0	0
Ethnicity				
Asian or Asian British	17	11.7	6	10.3
Black or Black British	47	32.4	21	36.2
Mixed	19	13.1	6	10.3
Other	9	6.2	3	6.8
White	45	31.0	18	31.0
Unknown/missing	8	5.5	3	5.2
Socio-economic status, IMD quintile by LSOA of home address				
1 (most deprived)	59	40.7	23	39.7
2	51	35.2	22	37.9
3	16	11.0	8	13.8
4	13	9.0	4	6.9
5 (least deprived)	6	4.1	1	1.7

CDOP, Child death overview panel; IMD, Index of multiple deprivation; LSOA, Lower layer super output area.

The relationship between the victim and perpetrator was not known or documented in almost half (47%, *n* = 27) of the cases. The perpetrator was known, but unrelated, to the victim in 23 (40%) of the deaths; six of the children (10%) were killed by a biological parent (in half of these cases, the child’s mother was also killed); and two children (3%) were killed by a stranger. Prior to their death, three quarters (74%, 43) of the children had been known to social services, with a third (33%) having active involvement from social services at the time of their death.

## 4 Results

In examining the casefiles, it was clear that many of the children had experienced forms of violence and harm in many ways, across multiple domains (Table 2). As the sample is of children who died because of violence, sadly none had escaped experiencing any form of violence.

**Table 2:** Children’s experiences of violence as victims and/or perpetrators.

Domain	Number of children (*n* = 58)	Percentage (rounded)
Child victims of violence/polyvictimization		
At home	39	67
At school	5	9
In the community	47	81
Victims (total)	58	100
Polyvictim (more than one domain)	34	59
Child perpetrators of violence/polyperpetration		
No recorded perpetration	24	41
At home	11	19
At school	16	28
In the community	22	38
Perpetrators (total)	34	59
Polyperpetrator (more than one domain)	19	33
Victim/Perpetrator overlap	34	59

The analysis below explores these different domains of violence and demonstrates how intervention models that separate victims and perpetrators failed to account for the overlap and co-occurrence of the violence the children experienced throughout their childhoods.

### 4.1 Violence at home: domestic violence, abuse and neglect

The majority of children (58%) in the sample had experienced childhood domestic violence and abuse in their home. Some (*n* = 3) of the children died in circumstances which related directly to an attack on their mother. None of the children received child-specific DVA support from a specialist service. In some ways this reflects the ubiquity of DVA in the lives of children, but it also highlights that there were missed opportunities to provide specialist, child-centered domestic violence support that would have been beneficial to the child at the time and which may also have affected the trajectory of their life. The casefiles showed that, when it was recognized, violence at home was dealt with via statutory safeguarding services and often led to a referral to child protection agencies, yet only four (7%) cases mentioned specific support referrals for DVA. Examination of the narratives in NCMD casefiles revealed three distinct but overlapping areas of concern. Firstly, although some mothers had raised concerns about the impact of DVA on their child, this was often missed by professionals:

*‘Young Person had attended numerous primary schools due to the family having to relocate under the police’s advice, when mother was in a relationship with a violent male. **It was thought that young person didn’t witness any violence**, but he himself displayed violent and threatening behaviour at primary school. The family moved into a refuge, and he then started a new school.’ (Emphasis added)*

Further, some parents believed that they had been able to shield their child from the violence *occurring* at home:

*‘[Child’s] mother told the review that she did not believe that he had been badly affected by domestic abuse because, for example, she made sure that the children did not witness any incidents.’*

This perspective implies that the child was somehow outside of the abusive dynamic and fails to acknowledge that the child’s own behaviour may reflect the violence experienced in their home. Overall, due to the framing of the mother as the primary victim, children were often not offered support for their victimization independently. It was apparent that children were not considered as direct victims of DVA themselves but were rather seen as witnesses, despite legislation specifically providing that ‘a child (under 18 years old) who sees, hears, or experiences the effects of domestic abuse and is related to the victim or the suspect is also to be regarded as a victim’ (Domestic Abuse Act, 2021). Secondly, onward support for families relied on the engagement and consent/permission of the non-abusing parent, and if this was not garnered then no further support was offered for the child.

*‘Mum has previously disclosed being a victim of domestic violence and it is likely he has witnessed some of this. Outcome of this referral - Mum was not willing to discuss the situation with the social worker or give consent for the SW to speak to school. The assessing SW **did not feel that threshold was met to override consent** … recommended the case to close … [Later entry on same case] Referral from Housing after the children’s mother disclosed [decades] of domestic abuse… Outcome: Referral for Early Help. In summary, there was a very brief period of intervention … which culminated in a withdrawal of consent by mother.’*

Here the support services relied on the mother’s consent for further sharing of information with school and the social workers. Due to the ‘threshold’ to override parental consent not being met, the case was closed, and the mother was signposted to ‘early help’ services (itself an oxymoron, after decades of abuse); the child was left unsupported and unrecognized. Furthermore, some of the casefiles identified additional structural issues that reduced support availability, including insecure immigration status of the non-abusing parent. Several parents of the children could not speak fluent English, and due to their recent migration may not have had a clear understanding of the child protection and policing systems in the UK, which may have impeded their ability to seek and engage with help and support. In addition to the limited availability of appropriate DVA child-specific support services, there was a further challenge in that, if services were offered, the children’s engagement was contingent on the compliance of their parents, as illustrated in the following case excerpts:

*‘[Child’s] parents **did not work well** with Children’s Services so [the child’s] voice was not really heard (emphasis added).’*

*‘Anonymous referral from a family member about parental acrimonious relationship with ongoing DA and threats to kill – mother disclosed some previous DA but **did not support** prosecution and case closed (emphasis added).’*

Lastly, the assessment of risk of harm was framed around the risks posed to the adult victim and thus failed to capture the longer-term risks to the child of further negative outcomes. There was an over-reliance on the Domestic Abuse, Stalking, Harassment and Honour Based Violence (DASH) risk assessment model, which uses metrics of frequency and severity to examine the risks of harm/murder as low/standard/high. The decision to rank risk as ‘standard’ resulted in no further action being taken in several cases. In one case, where the child was killed in a domestic homicide, there was evidence that the assessed risk had been graded as low/standard, resulting in no further action. In the case below it appears that professional judgement was influenced by the assumption that the child was only affected by the one incident they were perceived to have directly witnessed:

*‘Domestic violence on 3 occasions … Child was present only at the incident on [redacted] and a welfare check conducted. [Date] call received at school from concerned member of the public about the ongoing situation of domestic abuse and expressed concern for Child. … **All domestic were graded as medium or standard and the issues did not meet the criteria for Multi Agency Risk Assessment Conference (MARAC) referral due to the grading nor frequency** (emphasis added).’*

This highlights how the DASH risk-factor model fails to see the realities of living with DVA as significant by itself and warranting specialist support. This trend was repeated in other cases. The following case had been flagged due to nearly 40 local police notifications, yet ‘The incident was rated a medium and downgraded to standard risk assessment… the outcome was no referral required to children’s social care.’ In this case the processes for notification were in place via Operation Encompass, a scheme designed to enhance communication between police and the children’s school. However, in this and other cases, the scheme did not appear to result in child-specific support being offered, further demonstrating how the current risk model used to assess support needs for DVA does not work for children. Risk management strategies are based on the current and immediate risk of harm and death to the parent, which renders DVA as one of (potentially) many adverse childhood experiences for children that are not directly dealt with by specialist support services. At the time of writing similar concerns have been raised by the UK Government Safeguarding Minister Jess Phillips MP, and the DASH model is due to be reviewed for its suitability as a risk assessment model (Nathoo and Kraemer, 2025).

Eleven children (19%) had perpetrated violence at home, becoming a risk to other family members. Of these, two cases had previously assaulted their mothers, although this was sometimes framed in casefiles due to the inability of the mother to manage the child’s behaviour. As previously noted, some case records reflected a lack of connection being made between the child’s externalizing behaviour and the violence they were exposed to at home and elsewhere.

### 4.2 Violence at school and school exclusions

For some children school became a further domain of violence, either as a site of disclosure, victimization or perpetration. Of the cohort, 53 were of school age (aged 5 years or older); five (9%) children had been victims of violence at school, with 16 (30%) having been perpetrators of violence at school. The case records demonstrated that some children disclosed experiencing domestic violence and abuse to staff within their school, although this typically did not result in access to specialist child-focused DVA support, despite the risk to the child. However, it highlights the importance of trusting and supportive relationships between staff and at-risk pupils, and how schools could provide a potential route to providing children and young people with access to professionals without parental oversight. In two cases it was highlighted that the child disclosed physically intervening in the violent altercations, trying to protect their mother:

*‘[Child] reported bullying against him at school. The children witnessed domestic abuse on multiple occasions and must have been on alert to this at all times which will have been highly stressful. [Child] was directly involved on at least one occasion when he tried to protect his mum from violence by his dad. He reported to his class teaching assistant that dad was a bad man that does bad things to his mum.’*

For many children school was also a site of externalizing behaviours that may have been linked to the experience of violence at home, such as in the following case note, where a boy ‘seemed to be on a mission to turn people against him. He put on a “tough-man act” and deliberately walked and talked in an aggressive manner’. This indicates that some boys tried to appear tough or invulnerable in public spaces as a coping strategy to counterbalance victimization in private space. The case notes unusually acknowledged this as a way of dealing with harm, rather than simply seeing it as bad behaviour. When children engaged in school violence it often resulting in exclusion and/or a transfer to alternative educational provision. The casefiles reported that children were excluded due to *‘aggression’, ‘uncontrollable anger’, or ‘*emotional violent outbursts*’*. Schools’ inability to adequately support children who may ‘act out’ because of experiencing DVA was mentioned in some casefiles. Specifically, it was noted that the school dealt with the behaviour itself rather than viewing it as potentially linked to their adverse experiences; for example, with a school support plan critiqued as it ‘appears to be solely focused on the behaviour itself. There appears to be no consideration of why [child] was behaving in this way and if this could be as a result of what was happening at home*’.* This narrative was an enduring theme in the files, in that exhibiting violent and aggressive behaviour at school was seen in isolation of the child’s home circumstances. This is possibly related to the earlier point that children are rarely recognized as direct victims of DVA. However, displaying aggressive or violent behaviour at school often led to an onward referral to the Child and Adolescent Mental Health Service (CAMHS) with highlighted concerns around either mental health difficulties or possible neurodiversity.

### 4.3 Mental health and neurodiversity

Possible neurodiversity and/or concerns about learning difficulties/disabilities, speech and language difficulties or mental health difficulties were reported in the majority (78%) of the casefiles. However, onward referral to CAMHS was made only for 17 children, resulting in a diagnosis of a medical condition for 16 children (28%), and seven children (12%) being placed on an Education, Health and Care Plan (EHCP). Table 3 documents where children’s outward behaviour was noted in the casefiles as being related to a potential mental health or neurodiversity concern, and the fewer cases where a condition had been diagnosed. The high level of suspected cases indicates several potential issues. The first is whether professionals such as social workers and teachers are assigning labels prior to formal diagnosis or treatment. This presents a risk that some negative behaviours exhibited by young people are being attributed to health concerns rather than alternative causes, such as prior experiences of adversity and violence. The second issue is the distinction between those referred to CAMHS and those who were finally diagnosed again indicates that, at times, a CAMHS referral may not be appropriate and may point to the pathologization of children’s behaviour and the habitual referral to mental health support as a catch-all service. There are also issues with thresholds and attrition rates (as explored in the casefiles below), albeit noting that some of the children may have died while on CAMHS waiting lists.

**Table 3:** The extent of concerns about mental health and/or neurodiversity noted in the case records.

Mental health concern/neurodiversity (*n* = 58)	Suspected *n =* 58	Diagnosed *n =* 58	Cohort %
Either neurodiversity and/or mental health concern	29	16	78
Mental health concern	4	0	7
Neurodiversity	12	8	34
Conduct disorder	3	2	9
Learning difficulty/disability	9	4	22
Speech and language issues/referral	9	0	16
Onward referral to Child and Adolescent Mental Health Services	17	N/A	29
Education, Health and Care Plan	0	7	12

From the casefiles, it appeared that often the issues for which the children were being referred to CAMHS were quite broad and at times loosely attached to a specific concern related to mental health, rather than multiple forms of childhood adversity. For example, one case noted that:

*‘Child was referred to CAMHS on seven occasions, this included both external and internal referrals... [these] did not trigger follow ups or professional queries. Child, his mother and agencies reported concerns regarding child’s mental health over a [several] year period to CAMHS, however, none of these met the threshold for CAMHS intervention… It is deeply troubling and difficult to understand how a child who was a victim of stabbing, involved in a serious … incident … did not meet the threshold for CAMHS and his mental health was deemed moderate.’*

This narrative suggests that, although the child’s mental health was declining, CAMHS could not meet the child’s trauma-related needs and, indeed, were not the organization to which the referral should have been made. This appeared again in a further case, where a child was referred with concerns around conduct disorder, but also suggestions of vulnerability to exploitation. Again, whether CAMHS is an appropriate specialist support for concerns around exploitation is questionable:

*‘CAMHS referral; concerns around oppositional defiance, dysregulated arousal levels/ impulsivity and difficulty managing responses to frustrations, low self-esteem and validation seeking from peers through participating in risk-taking and physical aggression, difficulty adhering to parental boundaries and presenting with rather porous boundaries with peers; often putting their needs before his, placing him at risk of exploitation.’*

In another case a CAMHS referral was made due to the child’s behaviour at school, including being *‘intimidating’* and *‘*incredibly abusive verbally*’*. There is a growing body of research (Kewley et al., 2024) on the correlations between mental health and/or neurodiversity and involvement in offending behaviour, but the extent to which these concerns are compounded by exposure to private and/or public space violence is not yet known. Nonetheless, it appears that children are being referred to CAMHS due to concerns around victimization, aggression, and potential violence perpetration, arguably related to the lack of available specialist child-violence support services for professionals to refer to.

### 4.4 Violence in the community

The casefile analysis revealed that most children had experienced at least one form of violence in the community, separate from the school environment, either as perpetrator and/or victim, noting that due to the nature of the sample all children had experienced fatal violence. Forty-seven (81%) of the children had experienced violence in the community (i.e., not at home or school). Of these, almost half (46%) were viewed as being victimized within a broader context of serious youth violence, including child criminal exploitation or gang involvement. Twenty two (40%) of the children had also perpetrated violence in the community: one was being investigated for perpetrating a murder themselves, prior to their death; two case records mentioned intimate partner violence perpetration and sexual violence perpetration was mentioned in a further two cases (one case of sibling sexual abuse, and one of suspected of rape, although charges had not been laid). Knife carrying was mentioned in a quarter (14, 24%) of the case records.

Despite the potential harms caused to the children, most of those known to the criminal justice system were not provided with trauma-informed support but instead were referred to awareness raising style brief interventions, such as *‘*Choices and consequences*’*, which aim to educate children about the risks of exploitation and involvement in offending. This raises the question of whether children who exhibit signs of youth violence perpetration are being over-responsibilised for their behaviour, rather than being supported to overcome the factors underlying it. The most common intervention cited for children who were identified as ‘at risk’ of exploitation and involvement in public space violence was a brief educational intervention focused on their risk-taking behaviours. An example of how a focus on DVA was rerailed and focused on risks of SYV can be seen in the excerpt below:

*‘[Child] had been affected by domestic violence, having witnessed numerous incidents of violence perpetrated towards her by partners … [He] was subject to a Child Protection Plan … As part of the Child Protection Plan, the Social Worker visited [child] … to undertake direct work to help him understand risk from gang involvement and criminal exploitation.’*

One child had experienced knife-related domestic violence at home. This highlights the importance of knife-focused interventions not just being about the child’s own risks of victimization/perpetration in public space violence but also encompassing the experience of knife crime in private spaces. The role of education as both a prevention and intervention approach was repeatedly proposed by professionals throughout the casefiles. Often these interventions took the form of generic educational sessions, rather than being tailored to children who may have experienced violence at home or elsewhere, such as referrals to ‘[Knife Crime Awareness] project to elevate his insight into concerns about risky and unsafe behaviours and improve his resilience level to achieve better outcomes in his life’.

In addition to education and awareness raising approaches, there was also a repeated theme that children would be able to choose different pathways out of child criminal exploitation if only they were educated about the risks they were posing to themselves. For example, one child was spoken to about ‘the dangers of hanging around with older males … he was spoken to about the need to find suitable friends his own age’. If a child is subject to criminal exploitation, then the advice to seek friends their own age appears at best futile, at worst it places an undue responsibility for the child to mitigate against their own victimization. There were also several referrals made for children to youth violence education sessions of diverse forms. Two casefiles noted that the child had been referred to boxing interventions, although success was not ascertained as in one case they stopped engaging and the other was signposted only. No critical reflections in the casefiles of the suitability of boxing for child survivors of domestic violence were noted (Table 4).

**Table 4:** Children in conflict with the law.

Children in conflict with the law	Whole cohort *n* = 58	%
Known to the criminal justice system	20	34
Youth Offending Team involvement	14	24
Drug or alcohol misuse by child	13	22
SUSPECTED ‘gang-involved’	16	28
SUSPECTED Child exploited. Criminal exploitation	14	24
SUSPECTED Drug dealing by child	12	21
SUSPECTED Child exploited- sexual	3	5

## 5 Discussion

The findings reveal a fundamental systems failure in recognizing and responding to the complexity of children’s experiences of violence, particularly where polyvictimization and polyperpetration intersect. Children who move between, or simultaneously occupy, roles as both victims and perpetrators across home, school, and community domains are routinely overlooked by specialist services, leading to disjointed, duplicated, or entirely absent interventions. This is not a matter of attributing blame to children, but of identifying the many missed opportunities in which their behaviours, which may have indicated distress, went unrecognized and unsupported. Because agencies often categorized children’s experiences narrowly, and because professional assessments frequently diverged from family perspectives, crucial information failed to travel across systems. As a result, children’s overlapping experiences of harm were not made visible, preventing earlier and more effective intervention. These patterns underscore the urgent need for a holistic, child-centered approach capable of recognizing the full spectrum of violence that children navigate and responding in ways that address its root causes rather than its symptoms.

### 5.1 Inadequate current assessment frameworks for children experiencing and involved in violence

This study illustrates significant shortcomings in the assessment frameworks currently used to identify and support children experiencing violence, marginalization, and adversity. The casefiles indicate that widely used risk and adversity tools are often not fit for purpose, as they tend to prioritize historic or individualized ‘risk factors’ rather than capturing the complex, overlapping forms of violence that children may experience simultaneously across home, school, and community contexts. ACE-based screening models, for example, struggle to illuminate these concurrent patterns of harm. Moreover, children exposed to domestic violence and abuse were rarely recognized as victims in their own right or offered specialist support, reflecting an assessment culture that frequently narrows its focus to immediate risk. Social Services assessments routinely prioritized cases only when an imminent threat was identified and the perpetrator present, leaving many children without intervention despite persistent adversity. Similarly, the DASH risk assessment centers on mothers’ immediate risk of harm, overlooking the enduring impact of adult violence on children living within these environments. Indeed, recent research suggests that existing domestic violence risk assessment tools are generally inadequate in correctly identifying risk of homicide, leading the authors to advocate for a public health approach to domestic violence rather than a risk management model (Trood et al., 2026). Together, these practices reveal a systemic gap: the absence of a holistic, child-centered framework capable of assessing children’s experiences of violence independently of the non-abusing parent and with appropriate attention to both cumulative and long-term harm. Developing a dedicated child-focused domestic abuse risk assessment model is therefore urgently needed.

### 5.2 Enhancing children’s rights to direct support after domestic abuse as direct victims

Children must have a clear and unequivocal right to direct support following domestic abuse and perpetrators should be held to account for the harm they cause. Until children are fully recognized and treated as victims in their own right, as intended under the Domestic Abuse Act (2021), services for them will continue to be positioned as optional extensions of support offered to their non-abusing parents (most often mothers) rather than as essential interventions for them as individuals. The enduring barriers for children being recognized as direct victims have been highlighted in a recent joint targeted area inspections (JTAIs) (Ofsted et al., 2026). The inspection focused on children between 0 and 7 years old and found that, despite legislative changes, support for children is still largely unidentified, met with inconsistent responses, often lacking specialist local service provision, and too often centered on the adult’s needs over the children’s. This resonated with our findings.

While the 2025 UK VAWG Strategy’s allocation of £5.3 million for child survivors, alongside initiatives such as Family Hubs and a forthcoming trial of children’s interventions, is welcome, its emphasis on offering support *after* a perpetrator has left the family home risks leaving many children unprotected during periods of ongoing abuse, precisely when support is most urgently needed. Arguably we need to do more to support women and children when before they have left the perpetrator (Shrestha et al., 2026). Access to specialist help at times was constrained when parental consent is required, particularly when parents faced structural barriers such as poverty, insecure immigration status, or intensified scrutiny from professionals. There is established research as to why mothers may be reluctant to disclose and to seek help, much hinges on concerns around consequences of disclosure, intrusive intervention, and privacy concerns (Amel Barez et al., 2022; Haycock, 2025). In some cases, reluctance from non-abusing parents can be understood through the lens of *clientisation*, where mothers’ compliance is monitored more closely than children’s needs are addressed (Alberth and Bühler-Niederberger, 2015). Yet our findings point to the need for further exploration into how to balance children’s rights to support as direct victims of DVA, when parental consent for support is denied. We recommend further exploration into whether children should have a statutory, rights-based guarantee that entitles them to funded, timely, direct, specialist support.

### 5.3 Trauma-informed and gender transformative support for boys

Boys accounted for the vast majority of fatal stabbings within the NCMD cohort; violence prevention efforts must directly address how gendered expectations shape boys’ identities, coping strategies, and responses to trauma. There is a strong case for integrating trauma-informed and gender-transformative approaches within schools and youth interventions, particularly for boys who are disproportionately affected by serious youth violence (Zettler, 2021). Our findings indicated that schools were often early sites of disruptive behaviour and distress linked to unaddressed trauma, yet with limited opportunities for disclosure and narrow pathways to support. Trauma-informed educational models demonstrate the potential for schools to play a far more active role in recognising and responding to the effects of violence and adversity in children’s lives, offering relational, holistic, and context-aware environments (Public Health Wales 2026). Blower’s (2025) analysis of how education fails working-class boys emphasises the importance of approaches that address gendered pressures, structural inequalities, and the need to both challenge and affirm masculine identities, with his principles of belonging offering a clear framework for trauma-aware practice. An intersectional lens is essential, as research shows Black children in urban areas are less likely to be regarded by services as vulnerable and in need of support (Davis and Marsh, 2020). These dynamics intersect clearly with the pathways into serious violence: exclusion and movement into alternative educational provision, frequently noted in the casefiles, are associated with significantly heightened risks of involvement in violence within a year (Cornish and Brennan, 2025).

Emerging evidence supports engaging boys in gender-transformative, trauma-informed, and strengths-based programmes that encourage critical reflection on harmful norms while fostering emotional literacy, connection, and safer help-seeking (Snowdon et al., 2025). International evidence, such as school-based programmes in Australia, demonstrates that boys can benefit from structured spaces that promote communication and self-expression (Meads et al., 2025). UK policy direction is beginning to acknowledge this need, with the VAWG Strategy (UK Government 2025) committing to school-based violence prevention work with boys and piloted interventions for children showing early signs of violence. However, masculinity-focused sessions that focus on potential perpetration risk overlooking the realities of those simultaneously navigating unsafe homes, community violence, and/or criminal exploitation. For interventions to be genuinely gender-transformative and trauma-informed, they must support boys as both potential victims and potential perpetrators, with a focus on early identification and support.

### 5.4 Mental health, neurodivergence, and CAMHS as a catch-all referral route

CAMHS appeared to function as a ‘catch-all’ referral for behaviours linked to trauma from violence, but these were commonly framed as neurodiversity or mental health issues. Due to the nature of the casefiles, many children died before gaining an official diagnosis, so we are left not knowing whether their trauma was being mis-labelled, or whether they were just denied timely diagnosis and support. The potential over-identification of neurodiversity is of intense current debate. In December 2025 the UK Government ordered a review into evidence of overdiagnosis of ADHD and mental health problems (Department of Health and Social Care, 2025). Data indicates that rates of ADHD and autism diagnosis in the UK have risen from 15.5% of the 16–64-year-old population in 1993, to 22.6% in 2024 (ibid). However, clinicians have pushed back on this stating that concerns around overdiagnosis are divisive and discriminatory, with the reality being that ADHD is broadly underdiagnosed as compared with other high income countries (Thapar, 2026). A challenge is to disentangle the impact of untreated neurodivergence and mental ill health, which are cited in the NHS England ADHD taskforce report as, ‘educational failure, long-term unemployment, crime, substance misuse, suicide, mental and physical illness’ (NHS England 2025, p. 3) with these effects also being caused by other effects including unrecognized and untreated trauma. This is challenging to disentangle, as studies suggest that children who have been involved in violence are more likely to experience a mental health issue, and there is a high co-prevalence of neurodivergence among criminalized people (Kewley et al., 2024). However, the casefile analysis highlights that in many cases early experiences of domestic violence were not recognized and supported, and instead the child’s behaviours were then viewed as unrelated potential symptoms of mental health issues, an issue raised recently by the UK Domestic Abuse Commissioner (2025). These findings point to the need for greater professional curiosity about the prior experiences of violence that may sit beneath problematic behaviour in school. This mismatch led to poor engagement and limited impact. We need a conversation about whether the lack of consistently available specialist support for childhood violence in contributing to the over-pathologization of trauma.

### 5.5 Reframing risk: moving beyond criminality-focused interventions

Although 60% of the children went on to perpetrate violence, the remaining 40% did not, illustrating that trauma and adversity do not produce uniform behavioural outcomes. It is plausible that protective factors such as supportive relationships, stable schooling, or pro-social activities buffered some children from pathways into offending, although this was difficult to examine in the current dataset and requires further investigation. Crucially, many were primarily victims rather than perpetrators, and their vulnerabilities were expressed differently. This highlights that structural and interpersonal risks increase vulnerability but do not determine a child’s behaviour, exposure to violence shapes trajectories in diverse ways.

For the many children in the cohort who did perpetrate violence in school or community settings, the available interventions tended to focus narrowly on managing potential criminality rather than understanding their behaviour within the broader context of adversity. Despite clear evidence of violence occurring across multiple domains of their lives, referrals often centred on educating children about the risks posed by *their own* actions, effectively placing responsibility on them to avoid further victimisation. This offender-first orientation inadvertently responsibilised children for their involvement in youth violence and exploitation, while obscuring the structural and interpersonal harms they were experiencing. Consistent with the Youth Justice Board’s ‘Child First’ strategy, there is a pressing need to reorient practice towards holding adults and systems accountable and ensuring holistic, context-informed support that recognises the overlapping victim–perpetrator dynamics in many children’s lives (Case et al., 2023).

### 5.6 The systems gap for young people

Across these findings, a clear systems gap emerges. Current frameworks for identifying and supporting children experiencing violence are fragmented, crisis-driven, and largely adult-centric. Yet we know that violence is systemic and so our approaches need to manage to weave together disparate policy and practice landscapes (Adisa and Bond, 2024). Children’s experiences of abuse, adversity, and overlapping forms of violence across home, school, and community are routinely dealt with in siloes, with assessment tools focusing narrowly on immediate risk or parental harm rather than children’s long-term safety and wellbeing. As a result, children are left without timely, specialist, trauma-informed support, and schools, social care, and health services rely on inadequate or misaligned interventions that fail to address cumulative harm. This systemic failure creates avoidable gaps in care that can escalate risk and perpetuate cycles of violence.

## Data Availability

The data analyzed in this study is subject to the following licenses/restrictions: in England, statutory death notifications are required to be submitted to one of the 58 regional Child Death Overview Panels, for any child who dies before their 18th birthday. The Children Act 2004 mandates that the Panels must carry out a review of the circumstances of death and the modifiable factors relevant to the death as part of the child death review (CDR) process. Since 2019, this data has been collated by the NCMD Programme, allowing detailed analysis of the circumstances of death at a local and national level. Requests to access this data can be made to the NCMD. Requests to access these datasets should be directed to www.ncmd.info.
